# Immunization Catch-Up for Newly Arrived Migrants in France: A Cross-Sectional Study among French General Practitioners

**DOI:** 10.3390/vaccines9060681

**Published:** 2021-06-21

**Authors:** Sohela Moussaoui, Anne Marie Aurousseau, Sylvain Nappez, Julian Cornaglia, Gaylord Delobre, Sophie Blanchi, Louise Luan, Stéphanie Vandentorren, Olivier Bouchaud, Odile Launay, Louise Nutte, Roxane Liard, Mariela Skendi, Matthieu Mechain, Nicolas Vignier

**Affiliations:** 1 Department of Social Epidemiology, Institut Pierre Louis d’Épidémiologie et de Santé Publique (IPLESP), Sorbonne Université, INSERM, 75012 Paris, France; sohela.moussaoui@sorbonne-universite.fr (S.M.); Stephanie.VANDENTORREN@santepubliquefrance.fr (S.V.); 2 Department of Education and Research in General Medicine, Sorbonne Université, 75012 Paris, France; louisenutte@gmail.com (L.N.); roxane.liard@gmail.com (R.L.); mariela.skendi@gmail.com (M.S.); 3 Bordeaux University Hospital, Bordeaux University, 33000 Bordeaux, France; a-marie.aurousseau@hotmail.fr (A.M.A.); matthieu.mechain@gmail.com (M.M.); 4 Amiens University Hospital, 80000 Amiens, France; nappez.sylvain@chu-amiens.fr; 5 Perpignan Hospital, 66000 Perpignan, France; julinaglia@hotmail.com; 6 Louis Guilloux Network, 35000 Rennes, France; g.delobre@rlg35.org; 7 Le Mans Hospital, 72000 Le Mans, France; sblanchi@ch-lemans.fr; 8 Groupe Hospitalier Sud Ile-de-France, Department of Infectious and Tropical Diseases, 77000 Melun, France; louise.luan@gmail.com; 9 Assistance Publique Hôpitaux de Paris, AP-HP, Hôpitaux Universitaires Paris Seine-Saint-Denis, Hôpital Avicenne, Department of tropical and infectious disease, Université Sorbonne Paris Nord, UFR SMBH, LEPS, 93000 Bobigny, France; olivier.bouchaud@aphp.fr; 10 Société de Médecine des Voyages, 75013 Paris, France; 11 Assistance Publique Hôpitaux de Paris, AP-HP, Hôpital Cochin, Université de Paris, INSERM CIC 1417, F-CRIN I-REIVAC, 75014 Paris, France; odile.launay@aphp.fr; 12 Centre d’Investigation Clinique Antilles Guyane, CIC Inserm 1424, DRISP, Centre Hospitalier de Cayenne Andrée Rosemon, 97300 Cayenne, French Guiana, France; 13 French Collaborative Institute on Migration, ICM, CNRS, 93300 Aubervilliers, France

**Keywords:** family health, immigrants, transients and migrants, vaccine, catch-up

## Abstract

Background: Migrants often undergo an incomplete vaccination program in regards to the French recommendations. The aim of this study was to evaluate the practices of French General Practitioners’ (GPs) in terms of catch-up vaccination. Methods: A cross-sectional study was carried-out in 2017–2018 in France. An online questionnaire was disseminated by email through scholarly societies to GPs involved in the care and the vaccination of migrants. Analyses included univariate and multivariate analysis with a logistic regression model. Results: A total of 216 GPs completed the survey. A majority identified themselves with an average level regarding the prevention of infectious diseases among migrant populations (56.7%) and confirmed this is part of their daily practice (83.3%). The majority of respondents do not perform more than two injections on the same day. When compared to GPs working in health centres, those with a private practice are more likely to report returning to a full primary vaccination schedule (adjusted OR = 2.90, 95% CI [1.29–6.53]). Aside from the serology for hepatitis B and to a lesser extent for measles, other pre-vaccination serologies were not frequently used by GPs. When a migrant declares to be up-to-date with his immunisations, only 56.5% of doctors consider this information reliable. Conclusions: This study clarified the vaccination practices of GPs receiving migrant patients in consultation and showed its heterogeneity. An important need for benchmarks has been identified and these results were used for the elaboration of the French guidelines on vaccines catch-up.

## 1. Introduction

There is a reduced use of primary care services and disparities in terms of prevention care among the migrant population in France [1]. The absence of health insurance coverage reduces the probability of consulting a General Practitioner (GP) and can influence access to vaccinations [2]. Although no evidence suggests a link between migration and imported infectious diseases (except for tuberculosis), poverty is a risk factor and precarious migrants are exposed to higher risks of being infected by these diseases [3,4].

In Europe, outbreaks persist even in countries with a well-established vaccination program due to the presence of individuals who are not or incompletely vaccinated. In France, cases of vaccine preventable diseases such as measles [5,6] and chicken pox [5] have been reported, especially in migrant camps. Studies also suggest that vaccination coverage among immigrants is insufficient when compared to that of the host community [7,8,9,10,11]. The few existing data in France suggest a lower vaccination coverage among the migrant population than in the general population and does not meet public health objectives. Data collected from Doctors of the World in France (whose patient cohort was composed of 95% migrants, and one in three were in France for less than 3 months) show that around 20.0% of children aged up to 15 years old were not up to date for tetanus, diphtheria, or polio (TD-IPV) vaccines, 24.1% were not up to date for pertussis vaccine, and 28.1% were not up to date for the Measles-Mumps-Rubella (MMR) vaccine. [12]

In Europe, vaccination policies and practices are heterogeneous across countries [13,14]. Among individuals with uncertain or incomplete immunization status, three different types of vaccination strategies are used. The first is to vaccinate using a primary series of vaccines, as recommended by the ECDC [3] and the majority of countries who adopted vaccination guidelines for migrants [15,16,17]. This strategy guarantees the immunity but exposes the patient to a theoretical risk of reactogenicity. An alternative approach to systematic vaccination without immunization records is the use of serologic testing to verify the immune status and adapt the strategy. This second strategy avoids exposing the patient to side effects induced by the vaccine and it is held in high regard by some experts [18]. However, these serologies are not always reliable and are not reimbursed by the health insurance in France. The third strategy consists in considering the primary vaccination properly completed and focuses on the boosters in accordance with the national immunization program (NIP).

In this context of uncertainty, the objectives of the study were to describe the vaccination catch-up practices of GPs involved in the care of newly arrived migrants and to identify factors associated with each practice. 

## 2. Methods

### 2.1. Study Setting and Data Collection

A national cross-sectional study was carried out in France between April 2017 and September 2018. All physicians working with migrants’ care were eligible. The data was collected through an online self-administered survey disseminated by email to French physicians involved in the care and the vaccination of migrants. The invitation to take part in the survey was disseminated with the help of various partnerships across France including general practitioners’ groups, groups of doctors whose field of interest is infectious diseases or migrants’ health, the GP therapeutic training society research group (SFTG recherche), the primary care monitoring and research network Sentinelle, the national healthcare access centres collective (collectif PASS) and the non-governmental organisation (NGO) Médecins du monde network. Efforts were made to spread the survey to GPs with various types of practice and profiles in order to limit selection bias as much as possible. The study presented in this article was focused only on GPs who answered the survey, who were working in either a private practice, a medical centre, or in a “Health Care Access Point structure” (HCAP, Permanence d’Accès aux Soins de Santé). Most of the HCAPs are inside hospitals and provide both medical and social care to vulnerable populations, most of which lack health insurance. Answers were collected anonymously using www.wepi.org (accessed 18 June 2021), an Epiconcept^®^ website accredited to host personal health data by the shared healthcare information systems French agency (ASIP Santé). Filling out the form took an average of 20 min and all the answers were anonymous. We estimated that we sent the survey to around 1757 GPs through the different networks.

### 2.2. Population and Outcomes

The studied population was comprised of GPs practicing in France. Socioeconomical characteristics such as age, gender, type of practice and location of practice were collected. GPs were asked to define themselves as beginner, average or experienced with regard to the prevention of infectious diseases among migrant populations and whether this was part of their daily practice.

The survey was comprised of 15 questions addressing general situations about vaccination catch-up with regard to a migrant of 2-years-old or older without immunization records, reliability of the declarations, the number of injections administrated per day, and a question about the risk of hyperimmunization. Out of 15 questions related to vaccinations, 14 were multiple choice and one was an open question on the number of vaccinations administered daily. These choices were used to limit information bias.

### 2.3. Statistical Analysis

The outcome variables were carrying out a systematic full primary series of vaccines, the use of serology prior to the immunization, and the use of specialized websites informing about country-of-origin National Immunization Programs. Percentages were used to describe quantitative variables and a Chi2 test was used for comparisons between the three groups of GPs (those with a private practice, those employed in a medical centre, and those working in a HCAP), according to their gender, age, experience and daily practice with migrants.

Univariate and multivariate analysis using logistic regression models were performed for each practice. Variables included in the final models were those with a *p*-value <0.20, the age, and the gender. Missing data were excluded from the analysis model and were not imputed.

The analyses were performed using Stata 13.1 (Stata Corp., College Station, TX, USA).

### 2.4. Ethics and Regulation

Data were collected in a strictly anonymous manner with the participants’ consent collected online on the accredited website wepi.org. The collection of data has been subject to the individual information of participants, a privacy impact analysis and the study online deposit on the French Health Data Hub platform in accordance with the French and European General Data Protection Regulations. According to the French law, no ethical approval was required. 

## 3. Results

### 3.1. Characteristics of Participants

A total of 216 GPs responded to the survey (response rate 12.3%). Half of respondents (50.0%) had a private practice, 22.7% were employees of health centres, and 27.3% were practicing in HCAP structures.

The median age was 44.7 years, women represented 66.3% of the respondents, and 50.3% declared practicing in the Île-de-France region, which includes the city of Paris (*n* = 207 respondents).

In terms of experience with regard to the prevention of infectious diseases among migrant populations, 16.2% identified themselves as beginners (*n* = 210), 56.7% as “average”, and 27.0% as experienced. The identified level of experience was not different according to age. The majority (83.3%) felt experienced in migrant care.

### 3.2. General Questions

**Figure 1:**
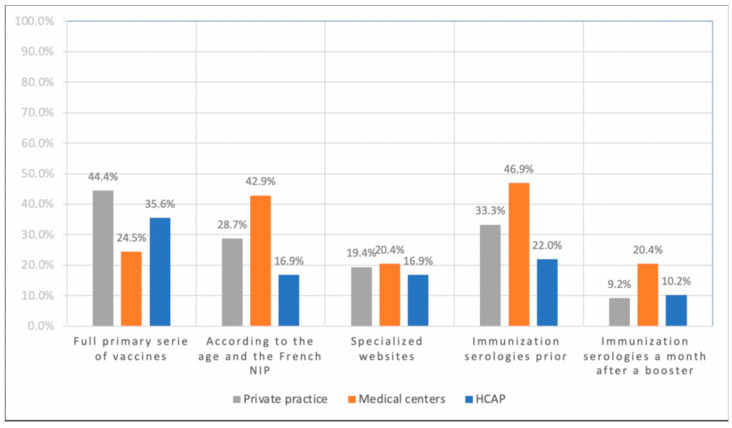
Vaccination strategy of 216 French general practitioners for an individual aged 2 and older without records of previous vaccinations (2017–2018).

The first question asked the respondent to reflect on the case of a child 2 years old or older without any record of previous vaccinations. In this situation, 37.5% stated they would carry out a full primary series of vaccines, and 28.7% declared they would carry on the immunization according to the age and the French NIP while considering that the patient had the primary series of vaccines (*n* = 216). Conversely, 19.0% reported adapting their prescription after consulting specialized websites about the country-of-origin’s NIP. Respectively 34.3% and 11.6% declared using immunization serologies prior to the vaccination or a month after a booster and adapting their prescription to the serologies. Figure 1 shows the answers according to the type of practice.

Regarding immunization catch-up for newly arrived migrants, 25.1% of GPs considered that there was a risk of hyperimmunization and 9.8% that hepatitis A immunization should be included.

**Figure 2:**
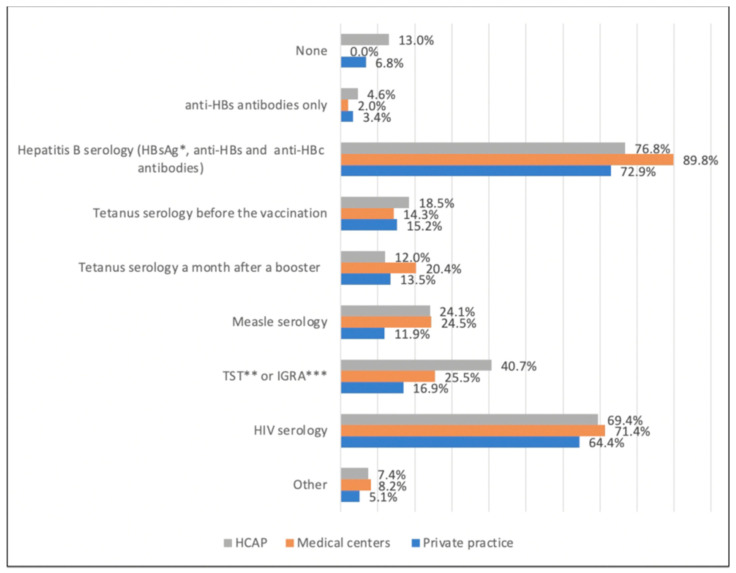
Use of pre-vaccinal serological test by 216 French general practitioners for a 35-year-old Syrian man (2017–2018). * HBsAg: hepatitis B virus surface antigen, ** TST: tuberculin skin test, *** IGRA: interferon-gamma-release assays.

With regard to the injections administered, 66.3% of GPs declared dispensing a maximum of two daily injections (Figure 2).

No differences in terms of age, gender, workplace, type of practice or experience were found for the number of daily injections administered.

### 3.3. Primary Vaccination

When using the logistic regression and after adjustment, a systematic and full primary series of vaccines was more often implemented by male than by female GPs (adjusted OR (aOR) = 1.88, *p* = 0.047, 95% confidence interval [CI], [1.01–3.51]). GPs exercising in a private practice were using this strategy more often than those employed by medical centres (aOR 2.90, 95% CI [1.29–6.53], *p* = 0.01) and those practicing in the Ile-de-France region more than those practicing outside it (aOR = 1.96, 95% CI [1.04–3.69], *p* = 0.036) (Table 1).

### 3.4. Serologies

When asked about the serologies for a 35-year-old male of Syrian origin, the majority answered using hepatitis B and HIV serologies, whereas the use of tetanus serology was low (Figure 2).

GPs aged less than 55-years-old declared using serologies prior to vaccination more often than those aged 55 and more (aOR 2.91, 95% CI [1.33–6.33], *p* = 0.007) and those working in HCAP less often than those with a private practice (aOR = 0.33, 95% CI [0.14–0.79], *p* = 0.013) (Table 2).

No significant statistical differences relevant to the age, gender, region of practice, type of practice, self-declared level of experience or daily practice was found for the use of serology a month after a booster.

### 3.5. Specialized Websites

After adjustment, women declared using specialized websites with information about country-of-origin NIP more often than men (aOR 2.92, 95% CI [1.20–7.09], *p* = 0.018) (Table 3).

### 3.6. Reliability of Declaration

When a patient or relative declared that the patient was up to date, 56.5% of GPs considered this response to be reliable and incorporated it into the vaccine strategy. Conversely, when either the patient or the relative declared that the patient was not up to date, 80.1% of the GPs considered this response to be reliable and carried out a full primary series of vaccines.

## 4. Discussion

### 4.1. Summary

This study clarified the vaccination practices of French GPs receiving migrants in consultation. It highlighted how a large proportion of GPs declared that the care of migrants was part of their daily practice despite the low proportion of GPs who considered themselves as experienced regarding the prevention of infectious diseases among this population. The study also suggests that the heterogeneity of vaccination catch-up practices are contingent on the GP’s type of practice.

### 4.2. Comparisons with the Existing Literature

#### 4.2.1. Vaccination Catch-Up Strategies

Many GPs, especially those having a private practice, declared carrying out a full primary series of vaccines, in the absence of records about the prior immune status. Countries such as Canada [15], Germany [16], the United Kingdom [17] and Australia [19] recommend this strategy in the absence of serological testing for the diphtheria, poliomyelitis and tetanus vaccines.

GPs with a private practice used this strategy more often than those working in health centers. These different approaches could be explained by a more difficult access to vaccinal serologies in private practice, which could limit their prescriptions. Moreover, the uses of serologies generally requires the existence of complete health coverage since it multiplies the out-of-pocket expenses for the patient who attends a series of consultations. For this reason, the approach that consists in avoiding the use of serologies, often financially inaccessible for a migrant population that lacks health care coverage, guarantees an efficient immunity despite exposing the patient to unnecessary vaccine doses and a theorical risk of hyperimmunization.

Some GPs considered that there was a risk of hyperimmunization by revaccinating migrants in France. For this reason, vaccine tolerance is a central concern and an important component of the immunization catch-up discussions for incomplete or unknown immunization status given that some vaccines can induce undesirable effects especially when they are given multiple times within close intervals. These risks include the Arthus’ reaction (type III hypersensibility reaction), which can occur with TD-IPV or TDaP-IPV (tetanus, diphtheria, acellular pertussis and inactivated poliovirus) vaccines even though the physiological existence is discussed, and its frequency misjudged [20]. Anaphylactic reactions [21] and peripheral neuropathy [22] have also been reported with tetanus vaccines as well as hypotonic-hyporesponsive episodes and febrile seizures with TDaP-IPV vaccine [23]. The risk of arm or leg local oedema after a supplementary dose is better characterized and seems frequent with high valence vaccines against diphtheria and pertussis [24]. In contrast, it seems that there is no risk to administer MMR, haemophilus type B, poliomyelitis, hepatitis B or meningococcus vaccines to a person already immune [25].

The experience and the frequency with which GPs encounter infectious diseases among migrant populations do not seem to be factors associated with vaccinal catch-up.

A great majority of GPs declared that they administer a maximum of two shots on the same day. According to experts, up to four or five shots can be performed on the same day, but caregivers should take into account the patient’s tolerance, especially when it comes to children [18,26]. Vaccines can be performed the same day or at any interval except for live viral vaccines that should be given either the same day or 4 weeks later.

#### 4.2.2. Serologies 

Although the use of serologies was part of GP practices, it mainly concerned the hepatitis B serology. This serology (HBs antigen, anti HBs and HBc antibodies) was offered before the hepatitis B vaccine by the majority of GPs. This matches recommendations for individuals originating from countries with a high prevalence of hepatitis B [18,26], as well as the position of countries such as Canada [18] and the US Centers for Disease Control and Prevention [27] for all new migrants originating from countries with a HBsAg prevalence of ≥2%. When the patient does not belong to a population at risk, most countries do not recommend the use of serological testing prior to hepatitis B vaccination because of the low risks associated with vaccinating individuals already infected with HBV against hepatitis B other than its inefficiency. In a Swiss study, the use of serologies 4 to 6 weeks after a vaccine against hepatitis B virus was assessed among 200 children without any vaccination records [28]. A booster-type antibody response was found in 81% of the children, indicating that younger age groups and migration from an urban area were significant determinants of the booster-type response.

In our study, the tetanus serology was not much used by GPs. The use of tetanus specific IgG antibody is both reliable and not expensive and its use is recommended in France 4 to 6 weeks after a dose of vaccine [18,26]. It can be assumed that a high level of tetanus antibodies indicates that the individual has also been vaccinated for diphtheria given that this vaccine is often administered in the same injection. Since the polio vaccine is still given orally in several countries, a tetanus immunity does not automatically mean that there is a good immunity for the poliomyelitis virus even though these vaccines are often given at the same time. The relevance of the immunochromatographic dipstick test (Tetanus Quick Stick) in immunization catch-up should be more precisely defined even though it use seems to be appropriate and cost-effective in situations where the immunization status is unknown [29].

#### 4.2.3. Reliability of Declarations Regarding the Immunization Status

In the absence of documentation or reliable records, the ECDC [3] and several countries recommend assuming the patient unimmunized and starting a catch-up program even if the patient says he was immunized [15,17,19]. The practices of one in two GPs therefore differ from these recommendations.

### 4.3. Strengths and Limitations

The major contribution of the study is the exposure it yields on how GPs undertake the vaccination catch-up of newly arrived migrants. These findings are relevant to General Practice because they show that depending on the GPs profile and type of practice, different approaches are used. These different approaches were taken into account in the elaboration of the National Guidelines. [26]

The proportion of respondent GPs exercising in a private practice or employed was comparable to the French population of GPs; however, women and young doctors were over-represented [30].

The main limitation of the study includes a selection bias. For our study, a convenience sampling was performed because it allowed us to rapidly gather information and it was a cost-effective method. Thus, our results cannot be generalized to all GPs in France because of the under-representation of some subgroups including GPs who do not receive migrants in their daily practice. Our sampling method also led to an insufficient power and probably involved difficulties in identifying differences between subgroups. The low participation rate of GPs outside of the Île-de-France region made the study less representative of GPs across France. This was probably due to difficulties encountered when disseminating the survey to networks outside the greater Parisian region. Nevertheless, the answers were probably from the most concerned GPs since there is a high concentration of newly arrived migrant populations in the Île-de-France region (38.2% of the total French immigrant population and 32.1% of those who arrived in France in the last 5 years live in the Parisian region) [31]. Another common shortcoming of web-based studies is the self-selection of study participants. Therefore, participants who answered were probably those who were the most interested in the topic because it was part of their practice. They were also probably more competent at the same time in the topic and in the use of new media than the rest of the targeted population.

## 5. Conclusions

GPs’ vaccination catch-up practices were heterogeneous and depended on their type of practice. Serologies were infrequently used apart from hepatitis B. The results of this study were drawn upon in the development of comprehensive national guidelines in France, in order to integrate the different vaccination practices as well as the difficulties encountered by GPs.

The dissemination and accessibility of websites and other relevant materials must be developed to support the vaccination catch-up strategies of general practitioners.

Targeting migrants for catch-up vaccination appears to be a cost-effective strategy when it includes social mobilization, vaccine programs and education campaigns [32]. It follows that vaccination catch-up programs for migrants should focus on developing these key areas. The implementation of epidemiological surveillance, the use of immunization electronic records as well as the development of a cross-border collaboration with the sharing of good practices and health data could together contribute to achieving the public health goals on vaccination coverage in migrant populations.

## Figures and Tables

**Table 1:** Results of logistic regression models estimating the association between carrying out a systematic full primary series of vaccines and general practitioner’s profile, among 219 general practitioners interviewed in France (2017–2018).

Covariates	Analysis			Univariate			Multivariate		
	*n*	%	*p*	OR	95% CI	*p*	aOR	95% CI	*p*
All	216	37.5					*n* = 204		
Gender	214								
Woman	142	31.7	0.016	ref.			ref.		
Man	72	48.6		2.04	[1.14–3.65]	0.016	1.88	[1.01–3.51]	0.047
Age	214								
Less than 55 years old	153	35.9	0.363	0.76	[0.41–1.38]	0.364	1.14	[0.58–2.27]	0.699
55 years old and more	61	42.6		ref.			ref.		
Region	205								
Île de France	104	43.3	0.087	1.64	[0.93–2.91]	0.088	1.96	[1.04–3.69]	0.036
Outside Île de France	101	31.7		ref.			ref.		
Type of practice	216								
Private practice	108	44.4	0.054	2.47	[1.16–5.24]	0.019	2.90	[1.29–6.53]	0.010
Health Center (employed)	49	24.5		ref.			ref.		
HCAP	59	35.6		1.70	[0.73–3.95]	0.214	2.33	[0.92–5.93]	0.075
Experience	210								
Beginner	34	41.2	0.736	1.40	[0.58–3.37]	0.452	/		
Average	119	37.8		1.22	[0.63–2.36]	0.563			
Experienced	57	33.3		ref.					
Daily practice	216								
Yes	180	36.7	0.572	0.81	[0.39–1.68]	0.572	/		
No	36	41.7		ref.					

ref.: reference category, OR: Odds Ratio, aOR: adjusted Odds Ratio, 95% CI: 95% Confidence interval, *p*: chi square *p*-value, HCAP: Health Care Access Permanency structures.

**Table 2:** Results of logistic regression models estimating the association between the use of serology prior to the immunization and general practitioner’s profile, among 219 general practitioners interviewed in France (2017–2018).

Covariates	Analysis			Univariate			Multivariate		
	*n*	%	*p*	OR	95% CI	*p*	aOR	95% CI	*p*
All	216	33.3					*n* = 213		
Gender	214								
Woman	142	38.0	0.034	1.98	[1.05–3.77]	0.036	1.61	[0.82–3.17]	0.162
Man	72	23.6		ref.					
Age	214								
Less than 55 years old	153	38.2	0.001	3.29	[1.55–6.98]	0.002	2.91	[1.33–6.33]	0.007
55 years old and more	61	16.4		ref.			ref.		
Region	205								
Île de France	104	31.7	0.885	0.96	[0.53–1.72]	0.885	/		
Outside Île de France	101	32.7		ref.					
Type of practice	216								
Private practice	108	33.3	0.024	0.57	[0.28–1.12]	0.105	0.68	[0.33–1.38]	0.285
Helth Center (employed)	49	46.9		ref.			ref.		
HCAP	59	22.0		0.32	[0.14–0.73]	0.007	0.33	[0.14–0.79]	0.013
Experience	210								
Beginner	34	35.3	0.407	0.81	[0.33–1.94]	0.631			
Average	119	30.2		0.64	[0.33–1.24]	0.185	/		
Experienced	57	40.3		ref.					
Daily practice									
Yes	180	33.9	0.699	1.17	[0.54–2.52]	0.703	/		
No	36	30.6		ref.					

ref.: reference category, OR: Odds Ratio, aOR: adjusted Odds Ratio, 95% CI: 95% Confidence interval, *p*: chi square test *p*-value, HCAP: Health Care Access Permanency structures.

**Table 3:** Results of logistic regression models estimating the association between the use of specialized websites informing about country-of-origin National Immunization Programs and general practitioner’s profile, among 219 general practitioners interviewed in France (2017–2018).

Covariates	Analysis			Univariate			Multivariate		
	*n*	%	*p*	OR	95% CI	*p*	aOR	95%CI	*p*
All	216	19.0					*n* = 213		
Gender	214								
Woman	142	23.9	0.012	2.92	[1.23–7.00]	0.016	2.92	[1.20–7.09]	0.018
Man	72	9.7		ref.			ref.		
Age	214								
Less than 55 years old	153	18.9	0.876	1.06	[0.49–2.29]	0.876	0.87	[0.39–1.93]	0.733
55 years old and more	61	18.0		ref.			ref.		
Region	205								
Île de France	104	16.3	0.321	0.70	[0.35–1.42]	0.323	/		
Outside Île de France	101	21.8		ref.					
Type of practice	216								
Private practice	108	19.4	0.888	0.94	[0.41–2.19]	0.892	/		
Health Center (employed)	49	20.4		ref.					
HCAP	59	17.0		0.79	[0.30–2.10]	0.703			
Experience	210								
Beginner	34	23.5	0.643	1.64	[0.56–4.76]	0.362	/		
Average	119	20.2		1.35	[0.58–3.12]	0.487			
Experienced	57	15.8		ref.					
Daily practice	216								
Yes	180	18.3	0.587	0.79	[0.33–1.88]	0.588	/		
No	36	22.2		ref.					

ref.: reference category, OR: Odds Ratio, aOR: adjusted Odds Ratio, 95% CI: 95% Confidence interval, *p*: chi square test *p*-value, HCAP: Health Care Access Permanency structures.

## Data Availability

All the relevant data for our analyses are fully described in the paper and can be made available on request. All data used for the analysis are available on request from the corresponding author.
